# Ferritin-Based HA DNA Vaccine Outperforms Conventional Designs in Inducing Protective Immunity Against Seasonal Influenza

**DOI:** 10.3390/vaccines13070745

**Published:** 2025-07-10

**Authors:** Hongzhe Lin, Yuxuan Jiang, Yan Li, Yiwei Zhong, Mingyue Chen, Weiyu Jiang, Rong Xiang, Najing Cao, Lei Sun, Xuanyi Wang, Lu Lu, Qiao Wang, Guangyue Han, Duan Ma, Bin Wang

**Affiliations:** 1 Key Laboratory of Metabolism and Molecular Medicine, Ministry of Education & Department of Biochemistry and Molecular Biology, Fudan University, Shanghai 200032, China; 21111010014@m.fudan.edu.cn (H.L.); duanma@fudan.edu.cn (D.M.); 2 Institutes of Biomedical Sciences, Fudan University, Shanghai 200032, China; 23111510056@m.fudan.edu.cn (Y.J.); llsun@fudan.edu.cn (L.S.); xywang@shmu.edu.cn (X.W.); 3 Shanghai Institute of Infectious Disease and Biosecurity, Shanghai 200032, China; lian.2002@163.com (Y.L.); yiweizhong@fudan.edu.cn (Y.Z.); 23211020003@m.fudan.edu.cn (M.C.); 21111010061@m.fudan.edu.cn (W.J.); 24111020038@m.fudan.edc.cn (R.X.); 19111010055@fudan.edu.cn (N.C.); lul@fudan.edu.cn (L.L.); wangqiao@fudan.edu.cn (Q.W.); 4 Key Laboratory of Medical Molecular Virology (MOE/NHC/CAMS), School of Basic Medical Sciences, Fudan University, Shanghai 200032, China; 5 Hebei Provincial Center for Disease Control and Prevention, Shijiazhuang 050021, China; 6 Advaccine Biopharmaceuticals Suzhou Co., Ltd., Suzhou 215000, China

**Keywords:** DNA vaccine, influenza, hemagglutinin (HA) antigen, immunogenicity, ferritin-based vaccine

## Abstract

**Background**: Influenza remains a persistent public health challenge due to antigenic drift and shift, necessitating vaccines capable of eliciting broad and durable immunity. Hemagglutinin (HA) antigen serves as the critical target for eliciting protective immune responses against influenza. DNA vaccines offer distinct advantages over conventional platforms, including accelerated development and induction of both humoral and cellular immune responses. **Methods:** To optimize HA antigen presentation, we designed and systematically compared the immunogenicity and protective efficacy of HA antigen display strategies—bacteriophage T4 fibritin (HA-Foldon) and ferritin-based virus-like particles (HA-Ferritin)—versus monomeric HA DNA vaccines against seasonal influenza viruses. **Results**: HA-Ferritin showed superior structural stability. All vaccines induced similar HA-specific antibody levels, but HA-Ferritin elicited higher neutralizing antibodies and stronger T cell responses. Upon challenge, HA-Ferritin and HA-Foldon protected mice from weight loss and reduced lung virus loads by 3.27 and 0.76 times, respectively. Monomeric HA provided limited protection, with only 40% survival and minimal viral or pathological reduction. **Conclusions**: The HA-Ferritin DNA vaccine demonstrated enhanced immunogenicity and protection, supporting structured antigen display as a promising strategy for influenza DNA vaccine development.

## 1. Introduction

Influenza viruses are classified into four types (A, B, C, and D), with influenza A and B viruses primarily responsible for seasonal epidemics, while C and D types typically cause milder infections in humans. Globally, it is estimated that influenza A and B viruses are responsible for 291,000 to 645,000 deaths annually [1], presenting an ongoing challenge to public health systems.

Since the development of the first influenza vaccine in the 1940s, vaccination has remained the cornerstone strategy for influenza prevention [2]. Currently, licensed influenza vaccines primarily include inactivated vaccines, live attenuated vaccines, and recombinant subunit vaccines. However, these vaccines are not universal; their efficacy depends on the specific strains recommended annually by the World Health Organization (WHO) based on circulating trends. Currently influenza vaccines typically contain three components: two influenza A strains (H1N1 and H3N2) and one influenza B strain (Victoria lineage) [3]. The production of conventional inactivated and live attenuated vaccines relies mainly on eggs and mammalian cell culture systems. During serial passaging, the viruses may undergo adaptive mutations, leading to antigenic changes that can reduce vaccine effectiveness. In contrast to conventional platforms, DNA vaccines offer distinct advantages, including not relying on live viruses and the ability to be rapidly designed via gene synthesis based on published sequences, enabling timely updates to antigenic components in response to emerging variants. Furthermore, compared with traditional vaccines that primarily rely on antibody-mediated immunity, DNA vaccines can induce not only robust antibody responses but also stronger T cell immune responses, potentially eliciting more comprehensive and durable immunity [4,5]. This provides a potential platform for developing broad-spectrum vaccines against diverse pathogens.

Most influenza vaccines employ hemagglutinin (HA) as the primary antigen to elicit protective immunity against influenza. As the major surface glycoprotein of influenza viruses, HA mediates viral entry into host cells by binding to sialic acid (SA) receptors via its receptor-binding site (RBS) [6,7]. Studies have demonstrated that the majority of neutralizing antibodies induced by either natural infection or vaccination specifically target epitopes within HA. Consequently, HA represents a critical target for rational vaccine design. Substantial efforts have been devoted to developing engineered HA immunogens with improved structural stability and enhanced capacity to induce potent neutralizing antibodies [8]. Among these efforts, trimerization motifs [9] and virus-like particle (VLP)-based platforms [10] that optimize HA antigen presentation have garnered significant attention.

In recent years, a universal strategy utilizing trimerization motifs (such as bacteriophage T4 fibritin and leucine zipper GCN4) to engineer immunogens has been proposed. These motifs effectively lock proteins in their prefusion conformation, which more closely resembles the native structure and better exposes critical neutralizing epitopes. This protein engineering approach has led to breakthrough progress in vaccine development against various infectious diseases including HIV [11], SARS-CoV-2 [12], influenza virus [13], and RSV [14]. Studies have demonstrated that trimerization motifs can stabilize recombinant HA in the correctly folded prefusion state [15]. Compared to monomeric proteins, vaccines containing trimerized structural domains elicit significantly higher neutralizing antibody titers and more robust protective immune responses in immunized animals [16]. Building upon the foundational work [16] demonstrating the advantages of trimerized domains over monomers, we sought to extend the approach to address a critical unmet need in seasonal influenza prevention.

Beyond trimerization strategies, substantial evidence indicates that VLPs are more efficiently recognized by the immune system than monomeric antigens, inducing potent humoral immune responses. Notably, ferritin, which self-assembles from 24 subunits into highly symmetrical spherical nanoparticles (20–50 nm in diameter) [17], closely mimics the spatial architecture of authentic viruses. Multiple strategies have been developed to display antigens on ferritin nanoparticles, including genetic fusion [18], chemical conjugation [19], and SpyTag/SpyCatcher systems [20]. Among these, genetic fusion represents the most convenient, rapid, and efficient approach, demonstrating promising immunogenicity in preclinical studies [21]. Research has shown that nanoparticle-displayed antigens exhibit superior stability compared to their monomeric counterparts, significantly enhancing antibody responses against target antigens [18]. This strategy offers the distinct advantage that orderly arranged multimeric antigens facilitate B cell receptor (BCR) cross-linking, thereby promoting B cell activation and the generation of high-quality antibodies [22]. The ferritin-based antigen display platform is currently being explored for combating multiple pathogens including RSV [23], influenza [22], and SARS-CoV-2 [24]. Consequently, the combined strategies of trimerization motif stabilization and VLP-based HA antigen presentation represent highly promising vaccine development approaches.

In this study, we designed three DNA vaccine candidates targeting the seasonal influenza HA antigen: (1) monomeric HA, (2) trimerization-motif-stabilized HA (HA-Foldon), and (3) ferritin-displayed HA nanoparticles (HA-Ferritin). Following in vitro transfection, non-reducing gel electrophoresis confirmed the trimeric conformation of both HA-Foldon and HA-Ferritin constructs. Electron microscopy (EM) and dynamic light scattering (DLS) analyses verified the successful assembly of HA-Ferritin into spherical nanoparticles of approximately 25 nm in diameter. We then evaluated the immunogenicity and protective efficacy of these vaccines in mice. Our results demonstrated that monomeric HA induced only limited neutralizing antibodies, whereas the HA-Ferritin vaccine elicited significantly higher neutralizing antibody titers against homologous seasonal influenza virus compared to the HA-Foldon vaccine. Following intranasal challenge with homologous virus, both the HA-Foldon and HA-Ferritin vaccines showed reduced lung viral loads and attenuated pathological damage compared to control groups. These data support HA-Ferritin as a promising vaccine candidate against influenza infection.

## 2. Materials and Methods

### 2.1. Cell Lines and Viruses

The Expi293F and MDCK cell lines were kindly provided by Professor Lu Lu. MDCK cells were maintained in DMEM medium supplemented with 10% fetal bovine serum (FBS; BI Israel BioSciences, Beit-Haemek, Israel) and 1% penicillin–streptomycin (Gibco, Grand Island, NY, USA) and cultured at 37 °C with 5% CO_2_. Expi293F cells were cultured in 293 CD05 medium (Shanghai OPM Biosciences Co., Ltd., Shanghai, China) under agitation at 105 rpm, 37 °C, and 5% CO_2_.

Influenza A virus (A/Puerto Rico/8/1934 H1N1), kindly provided by Professor Lu Lu, was propagated in 9-day-old specific-pathogen-free embryonated chicken eggs (Boehringer Ingelheim, Beijing, China) by allantoic cavity inoculation. After 72 h incubation at 35 °C, allantoic fluid was harvested and clarified by centrifugation at 6000× *g* for 10 min. The crude viral stock was aliquoted and stored at −80 °C.

### 2.2. Animals

Female BALB/c mice aged 6–8 weeks were purchased from Beijing Vital River Laboratory Animal Technology Co., Ltd. (Beijing, China). All animal experiments were approved by the Institutional Animal Care and Use Committee of the Basic Medical Sciences, Fudan University (Ethics Approval No. 20230301-39). Mice were housed under controlled temperature and humidity conditions with ad libitum access to food and water.

### 2.3. Plasmid Design

The HA construct was designed based on the study by Masaru Kanekiyo et al. [25], incorporating the HA1-521 domain. This domain retains the native viral signal peptide at its N-terminus to enable secretory expression in eukaryotic systems, with a C-terminal 6×His tag added for purification. For HA-Foldon and HA-Ferritin constructs, the Foldon trimerization motif (derived from Fibritin, PDB: 4NCV_A) or *Helicobacter pylori* Ferritin (residues 5–167, GenBank: WP_000949190.1) was fused to the C-terminus of HA via a GGGS linker. All designs were validated by structural prediction using AlphaFold2, with results visualized in PyMOL.

Influenza HA genes (A/Wisconsin/67/2022 [H1N1], GenBank: WBO08838.1; A/Massachusetts/18/2022 [H3N2], UUV89790.1; B/Austria/1359417/2021 [B/Victoria lineage], XBP28073.1; A/Puerto Rico/8/1934 [H1N1], PDB: 6WCR_A) and Ferritin sequences were codon-optimized for human expression (including an added Kozak sequence) and cloned into the pVAX1 vector (synthesized by GenScript).

### 2.4. Plasmid Preparation

Constructs were transformed into DH5α competent cells. After verification by Sanger sequencing, bacterial stocks were stored at −80 °C. Plasmid DNA was extracted using an endotoxin-free maxiprep kit (Magen, Shanghai, China).

### 2.5. Protein Expression and Purification

Expi293F cells were transfected at a density of 2 × 10^6^ cells/mL using PEI transfection reagent (Shanghai Life-iLab Biotech, Shanghai, China; Cat. #AC04L092). Culture supernatants were harvested by centrifugation at 10,000× *g* for 30 min at 4 °C five days post transfection. For HA-His and HA-Foldon purification, the supernatants were subjected to affinity chromatography using Ni Sepharose Excel resin (Cytiva, Marlborough, MA, USA), involving sequential washing with 10 mM imidazole and elution with 250 mM imidazole. The eluted proteins were concentrated using 30 kDa centrifugal filters (Merck Millipore, Burlington, MA, USA) at 2000× *g* (4 °C) through multiple centrifugation cycles until reaching the desired volume, followed by buffer exchange into PBS. HA-Ferritin was purified by size-exclusion chromatography on a Superose 6 Increase 10/300 column (Cytiva, Marlborough, MA, USA) using an ÄKTA pure system (Cytiva, Marlborough, MA, USA).

### 2.6. PNGase F Treatment

Protein samples were denatured in a Denaturing Buffer at 100 °C for 10 min, followed by incubation with Glyco Buffer containing 10% NP-40 and PNGase F (New England Biolabs, Ipswich, MA, USA; Cat. #P0704) at 37 °C for 60 min to complete deglycosylation.

### 2.7. Polyacrylamide Gel Electrophoresis (PAGE)

Protein samples were separated using 4–12% Bis-Tris gels (GenScript Biotech Corporation, Nanjing, China) for both SDS-PAGE and Native PAGE analysis. For SDS-PAGE, samples were mixed with 5× loading buffer (Beyotime Biotechnology, Shanghai, China), heated at 100 °C for 10 min, and electrophoresed in MOPS buffer at 160 V. Native PAGE samples were prepared with non-denaturing/non-reducing loading buffer (Wansheng Haotian Biotechnology, Shanghai, China). Following electrophoresis, gels were stained with Coomassie Blue for 5 min with gentle agitation, destained with water, and imaged using a gel documentation system (Tanon Science and Technology, Shanghai, China, model mini space 2000).

### 2.8. Western Blot Analysis

After electrophoresis, proteins were transferred to PVDF membranes using a semi-dry transfer system (GenScript Biotech Corporation, Nanjing, China, eBlot L1). The membranes were blocked with 5% skim milk at 25 °C for 1 h, incubated with HRP-conjugated anti-His-tag monoclonal antibody (Proteintech Group, Wuhan, China, Cat. #HRP-66005) at 4 °C for 16–18 h, washed with PBST, and detected using an enhanced chemiluminescence substrate. Images were acquired using an eBlot touch imager (GenScript Biotech Corporation, Nanjing, China).

### 2.9. Dynamic Light Scattering (DLS)

Protein samples were centrifuged at 10,000× *g* for 10 min at 4 °C to remove aggregates, and the supernatants were filtered through 0.22-μm membranes. For analysis, 40 μL of each sample was loaded into a microcuvette, and particle size distribution was measured at 25 °C using a Zetasizer Nano Series instrument (Malvern Panalytical, Malvern, UK, ZS90) with three sequential scans per sample.

### 2.10. Transmission Electron Microscopy (TEM)

Protein samples (0.03 mg/mL) were applied to 300-mesh carbon-coated copper grids, freshly glow-discharged in argon. After 1 min incubation, excess liquid was blotted with filter paper, followed by three sequential 10 s stains with heavy metal salt solution and final blotting. Images were acquired using a Talos L120C transmission electron microscope equipped with a CCD camera.

### 2.11. Immunization and Challenge Experiments

Female BALB/c mice (6–8 weeks old) were immunized intramuscularly with 50 μL of plasmid DNA (25 μg) using a prime-boost strategy (days 0 and 21), followed by electroporation at the injection site (Inovio, Plymouth Meeting, PA, USA, OpBlock 0079 program). To evaluate the immunogenicity of trivalent HA-Ferritin vaccines, two additional groups received either equal-dose trivalent HA-Ferritin plasmids (25 μg per HA subtype) or low-dose trivalent HA-Ferritin plasmids (8 μg per HA subtype). Serum was collected via facial vein bleeding on days 21 and 35 post immunization. On day 35, mice were intranasally challenged with 20 μL of 10 LD_50_ A/Puerto Rico/8/1934 (H1N1) virus, and body weight was monitored for 14 days, with humane endpoint criteria set at ≥20% weight loss.

### 2.12. Enzyme-Linked Immunosorbent Assay (ELISA)

Antigen immobilization was performed by coating high-binding 96-well plates (Corning, New York, NY, USA) with target proteins at 2 μg/mL in carbonate buffer (pH 9.6) for 16 h at 4 °C. After three washes with PBST, plates were blocked with 5% skim milk at 37 °C for 1 h. Serum samples were then subjected to two-fold serial dilutions in 2% skim milk and incubated with immobilized antigens for 90 min at 37 °C. Following three washes with PBST, plates were incubated with HRP-conjugated goat anti-mouse IgG (H + L) (Thermo, Waltham, MA, USA, Cat. #C31430100), rat anti-mouse IgG2a (Southern Biotech, Birmingham, AL, USA, Cat. #1155-05), or rat anti-mouse IgG1 (Southern Biotech, Birmingham, AL, USA, Cat. #1144-05) antibodies at 37 °C for 1 h. Immunoreactivity was visualized using 3,3′,5,5′-tetramethylbenzidine (TMB) substrate (TianGen, Beijing, China) for precisely 15 min at room temperature. The enzymatic reaction was terminated by acidification with 1 M sulfuric acid and absorbance was measured at 450/620 nm using a microplate reader (Bio-Rad, Hercules, CA, USA).

### 2.13. Hemagglutination Inhibition (HI) Assay

Mouse serum was incubated with receptor-destroying enzyme (RDE, Denka Seiken Co., Ltd., Tokyo, Japan, Cat. #340122) at 37 °C for 18 h, followed by heat inactivation at 56 °C for 30 min. To remove nonspecific agglutinins, the treated serum was absorbed with guinea pig red blood cells (RBCs) in PBS at 4 °C for 1 h. Following centrifugation at 3000× *g* for 10 min to remove cellular debris, the supernatant was subjected to serial two-fold dilutions in U-bottom 96-well plates. Each diluted serum sample was mixed with 4 hemagglutination units of virus and incubated at 25 °C for 30 min to allow antibody–virus interaction. HI was then assessed by adding 1% guinea pig RBC suspension and incubating at 25 °C for 40 min. The HI titer was determined as the reciprocal of the highest serum dilution that completely inhibited hemagglutination.

### 2.14. Enzyme-Linked Immunospot (ELISPOT) Assay

Spleens were aseptically harvested from euthanized mice at day 35 post booster immunization and mechanically dissociated into single-cell suspensions. Erythrocytes were lysed using ammonium chloride buffer to isolate lymphocytes. For antigen-specific stimulation, 1 × 10^6^ cells/well were plated in mouse IFN-γ pre-coated ELISPOT plates (Dakewe Biotech Co., Ltd., Shenzhen, China, Cat. #2210005) with 2 μg/well of strain-matched peptides (H1N1: FERFEIFPK, SVSSFERFEIFPK; H3N2: SYNAELLVAL, AELLVALEN) and cultured for 16 h at 37 °C with 5% CO2. Following incubation, plates were washed with PBST and then incubated with biotinylated detection antibody at 37 °C for 1 h. After five washes with PBST, plates were incubated with streptavidin-HRP conjugate at 37 °C for 1 h, followed by PBST washes and incubation with AEC substrate at 25 °C for 10 min. Spot formation was quantified using an automated ELISPOT reader (AID GmbH, Strassberg, Germany).

### 2.15. Quantitative Real-Time PCR (qPCR)

Viral load quantification was performed using absolute qPCR with a plasmid standard containing the M2 gene of A/Puerto Rico/8/1934 (H1N1). Viral RNA was extracted from lung homogenates using a commercial RNA isolation kit (Sparkjade, Jinan, China), reverse-transcribed into cDNA, and amplified with specific primers (forward: 5′-GACCAATCCTGTCACCTCTGAC-3′; reverse: 5′-AGGGCATTTTGGACAAAGCGTCTA-3′) on a Real-Time PCR System (Bio-Rad Laboratories, Hercules, CA, USA, Model CFX96). The PCR protocol included an initial denaturation step at 95 °C for 30 s, followed by 40 cycles of denaturation at 95 °C for 10 s and annealing/extension at 60 °C for 30 s.

### 2.16. Histopathological Analysis

Lung specimens were processed for comprehensive histopathological evaluation. Tissue samples were initially fixed in 4% paraformaldehyde for 48 h, subsequently embedded in paraffin, and sectioned at 4-μm thickness for histological staining. Parallel sections were prepared for hematoxylin–eosin (H&E) staining to assess inflammatory infiltration. Stained sections were imaged using an EVOS AMG inverted fluorescence microscope (Advanced Microscopy Group, Bothell, WA, USA). Pathological scoring (1–5 scale) was performed as follows: Grade 1, no significant lesions; Grade 2, <25% lobar area with perivascular/parenchymal infiltration; Grade 3, 25–50% involvement; Grade 4, 50–75% involvement; and Grade 5, >75% involvement.

### 2.17. Statistical Analysis

Data were analyzed by one-way/two-way ANOVA with Tukey’s post hoc test. Values are presented as mean ± SEM (*n* = 5 biological replicates per group). Statistical significance was defined as *p* < 0.05. All analyses were conducted with GraphPad Prism software. (version 9.0; GraphPad Software, San Diego, CA, USA)

## 3. Results

### 3.1. Design and Characterization of Influenza HA Constructs

**Figure 1:**
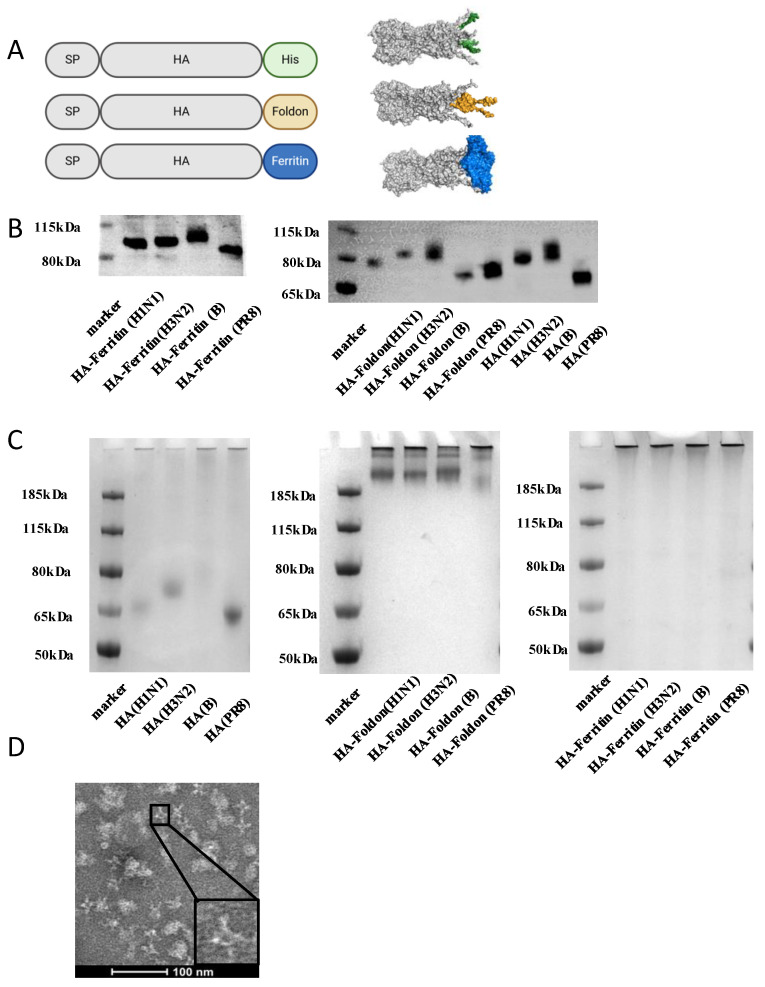
**Vaccine designs and characterizations of influenza HA constructs in vitro.** (**A**) Schematic illustration of HA, HA-Foldon, and HA-Ferritin vaccine designs and in vitro assembly. HA is the major surface antigenic protein of influenza virus. HA-Foldon was constructed by fusing HA with the bacteriophage T4 fibritin trimerization motif to stabilize its prefusion conformation. HA-Ferritin displays HA antigens on ferritin nanoparticles, which self-assemble into spherical structures (~20–50 nm in diameter) composed of 24 subunits, thereby enhancing immunogenicity. (**B**) Expression analysis of vaccine antigens by Western Blot. Plasmids encoding influenza HA antigens were transfected into Expi293F cells. At day 5 post transfection, cell supernatants were collected and mixed with SDS-containing loading buffer, followed by denaturation at 100 °C for 10 min. Protein expression was detected using an anti-His monoclonal antibody to confirm successful antigen expression and compare expression levels. (**C**) Oligomeric state analysis by Native PAGE under non-reducing conditions. Cell supernatants containing HA and HA-Foldon were purified via His-tag affinity chromatography, while HA-Ferritin was purified by SEC. The purified antigens were then analyzed by Native PAGE to verify the formation of higher-order oligomers. (**D**) TEM imaging of SEC-purified HA-Ferritin. The morphology and size of HA-Ferritin nanoparticles were directly visualized by transmission electron microscopy (TEM). Scale bar: 100 nm.

To develop structurally optimized influenza vaccine candidates, we utilized structure-based design principles to engineer three hemagglutinin (HA) immunogens derived from the soluble ectodomain (lacking the transmembrane domain) sequences representing contemporary seasonal strains (A/Wisconsin/67/2022 (H1N1), A/Massachusetts/18/2022 (H3N2), and B/Austria/1359417/2021 (B/Victoria)) and the prototype H1N1 PR8 strain. The constructs incorporated distinct C-terminal modifications to modulate antigenic architecture: (1) native HA (untagged soluble ectodomain); (2) HA-Foldon, ectodomain fused at the C-terminus to the bacteriophage T4 fibritin trimerization motif (Foldon) to enforce trimeric assembly; and (3) HA-ferritin, ectodomain fused with self-assembling ferritin nanoparticles for multivalent display. All constructs included C-terminal hexa-histidine tags for purification (Figure 1A).

AlphaFold2 structural predictions confirmed that all constructs maintained the canonical HA trimeric conformation, though with distinct stabilization profiles. Notably, the HA-Ferritin exhibited superior structural rigidity, particularly in its stem/base region (Figure 1A). To evaluate protein expression, Expi293F cells were transiently transfected with each plasmid construct. Expression in Expi293F cells yielded constructs with differential migration patterns; HA-Ferritin exhibited the highest molecular weight, followed by HA-Foldon and then HA by Western blot (Figure 1B), all of which migrated at sizes larger than their theoretical predictions, suggesting post-translational modifications. Deglycosylation with PNGase F treatment reduced molecular weights to match unglycosylated HA (Figure S1C), confirming N-linked glycosylation as the primary modification. Native PAGE showed HA-Foldon and HA-Ferritin as high-molecular-weight complexes (>185 kDa), consistent with trimeric assembly, whereas native HA remained monomeric (Figure 1C). Size-exclusion chromatography (SEC) further distinguished the constructs, with HA-Ferritin eluted earlier (Figure S1B), indicative of large particle sizes. Transmission Electron Microscopy (TEM) revealed that HA-ferritin formed uniform 24.195 ± 3.513 nm (mean ± SD) spherical nanoparticles displaying surface-exposed protruding HA spikes (Figure 1D), while dynamic light scattering (DLS) showed size distributions of 50–500 nm for HA-Ferritin versus 0.5–10 nm for soluble HA/HA-Foldon (Figure S1A). These results demonstrate the successful generation of HA immunogens, structurally distinct HA antigens with increasing levels of organization, from monomers to trimeric nanoparticles. This hierarchy of antigenic architectures provides a platform to interrogate structure–function relationships in eliciting immunogenicity in each design.

### 3.2. Humoral Immune Responses Following Vaccination

**Figure 2:**
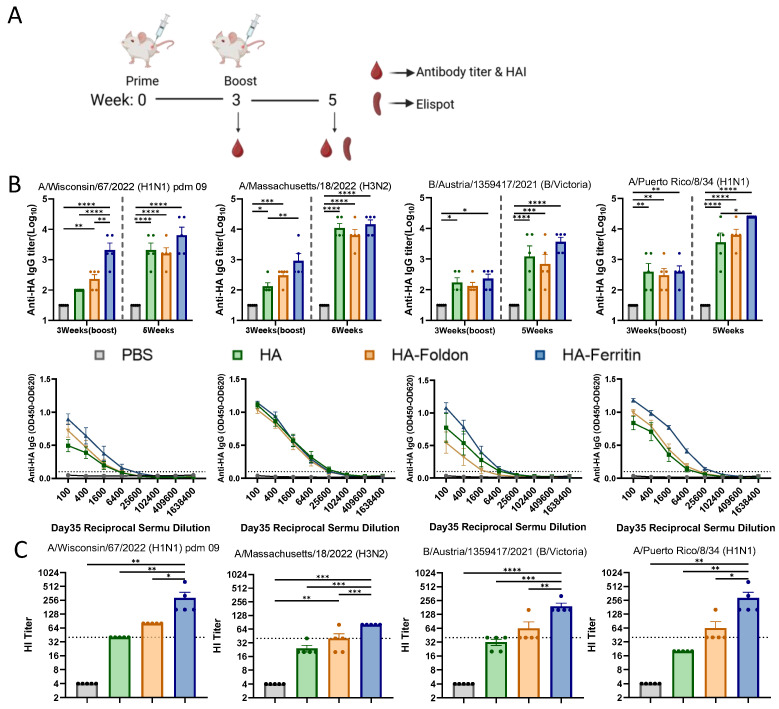
**Humoral immune responses following vaccination.** (**A**) Immunization schedule. BALB/c mice (*n* = 5 per group) were intramuscularly immunized with 25 μg of each antigen-encoding plasmid delivered via electroporation, followed by booster immunization on day 21. Blood samples were collected immediately before and two weeks after the booster immunization for serological analysis. Spleens were harvested for subsequent cellular immune response analysis. (**B**) HA-specific IgG antibody titers measured by an ELISA in serum samples collected immediately before and two weeks after the second immunization. Dotted lines indicate the seropositivity threshold (OD450 = 0.1), with values above this cutoff considered positive. (**C**) Hemagglutination inhibition (HI) titers against the homologous viral strain measured two weeks post boost immunization. Dotted lines represent the HI titer of 40, the threshold for protective immunity. Data points represent individual animals. Values are expressed as mean ± SEM. Statistical significance was determined by two-way ANOVA (**B**) or one-way ANOVA (**C**) with the following thresholds: ns, not significant; * *p* < 0.05; ** *p* < 0.01; *** *p* < 0.001; and **** *p* < 0.0001.

To evaluate the immunogenicity and protective potential of DNA vaccines encoding seasonal influenza strains (H1N1, H3N2, and B) and the prototypic PR8 strain, female BALB/c mice were immunized via intramuscular injection followed by electroporation twice at 25 μg of each monovalent antigen plasmid per dose administered in 21-day intervals (Figure 2A), while a PBS-injected group served as the negative control. Serum samples were collected at designated time points, and HA-specific antibody levels were measured by an ELISA. Following primary immunization (Day 21), all vaccine groups exhibited significantly elevated anti-HA antibody responses against their respective antigens compared to the control group. After the booster immunization (Day 35), the antibody responses further increased, with all monovalent antigen groups inducing significantly higher anti-HA antibody titers than the negative control (Figure 2B). Although no statistically significant differences were observed between the HA-Ferritin group and the HA or HA-Foldon groups, the HA-Ferritin group consistently demonstrated a trend toward higher antibody titers across all influenza subtypes. Analysis of serum IgG isotypic profiles after the second immunization revealed that all vaccinated groups developed significantly higher titers of both IgG1 (Th2-associated) and IgG2a (Th1-associated) antibodies compared to the PBS-treated control (Figure S2). While the magnitude of response varied slightly between vaccine groups, these differences did not reach statistical significance, suggesting similar Th1/Th2 polarization patterns across all formulations.

Hemagglutination inhibition (HI) antibody titers measured at day 35 post boost immunization revealed significant differences among vaccine groups across influenza strains (Figure 2C). Geometric mean titers (GMTs) demonstrated the following patterns: for the H1 subtype, HA, HA-Foldon, and HA-Ferritin groups showed 40, 80, and 242.5, respectively; H3 subtype, 22.97, 34.82, and 80; B subtype, 30.31, 52.78, and 183.79; and PR8 strain, 20, 52.78, and 242.51. Statistical analysis indicated that only the HA-Ferritin group exhibited significant differences compared to other vaccinated groups, while no significant difference was observed between HA and HA-Foldon groups. Notably, the HA-Ferritin group induced protective relevant HI titers (HI titer > 40) against all tested strains, followed by the HA-Foldon group, which reached protective thresholds for the H1 and B subtypes as well as for the PR8 strain, whereas the HA group only showed protective potential against the H1 subtype. These results demonstrate that the ferritin nanoparticle-based vaccine design significantly enhances neutralizing antibody production, exhibiting outstanding potential for influenza vaccine development.

### 3.3. T Cell Immune Responses Following Vaccination

**Figure 3:**
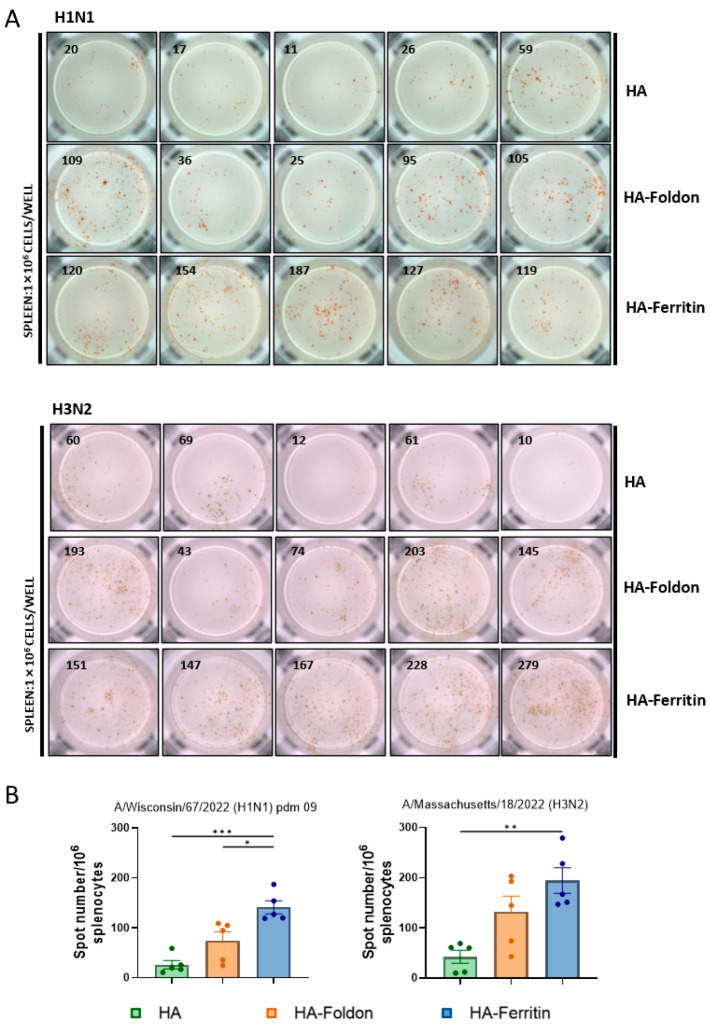
**T cell immune responses following vaccination.** Mice were immunized with monovalent seasonal influenza vaccines based on H1N1 and H3N2 strains. Two weeks after the booster immunization, spleens were collected and splenocytes (1 × 10^6^ cells per well) were stimulated in vitro with H1 or H3 antigenic peptides. IFN-γ-secreting cells were detected using an ELISPOT assay after incubation with anti-IFN-γ antibody. (**A**) Representative ELISPOT images showing spot-forming units (SFUs), with each image representing results from an individual mouse. (**B**) Quantification of IFN-γ-secreting lymphocytes in splenocytes. Data points represent the number of IFN-γ-secreting cell clones per 1 × 10^6^ splenocytes from individual animals (*n* = 5). Data are presented as mean ± SEM. Statistical significance was determined by one-way ANOVA: ns, not significant; * *p* < 0.05; ** *p* < 0.01; and *** *p* < 0.001.

Since DNA vaccinations are expected to elicit T cell-mediated immune responses, we quantified IFN-γ-secreting lymphocytes in splenocytes using an ELISPOT assay following peptide stimulation in vitro at day 35 post booster immunization. The HA-Ferritin vaccine induced significantly stronger IFN-γ responses, with 5.32-fold and 1.91-fold greater lymphocyte activations for H1N1, and corresponding increases of 4.58-fold and 1.48-fold for H3N2 compared to that of the HA-Foldon or HA groups, respectively (Figure 3A,B). These data reveal substantial differences in antigen-specific T cell responses induced by structurally distinct DNA vaccines, with the HA-Ferritin construct demonstrating markedly superior cellular immunogenicity.

### 3.4. Protective Efficacy of Vaccination Against Homologous Viral Challenge

**Figure 4:**
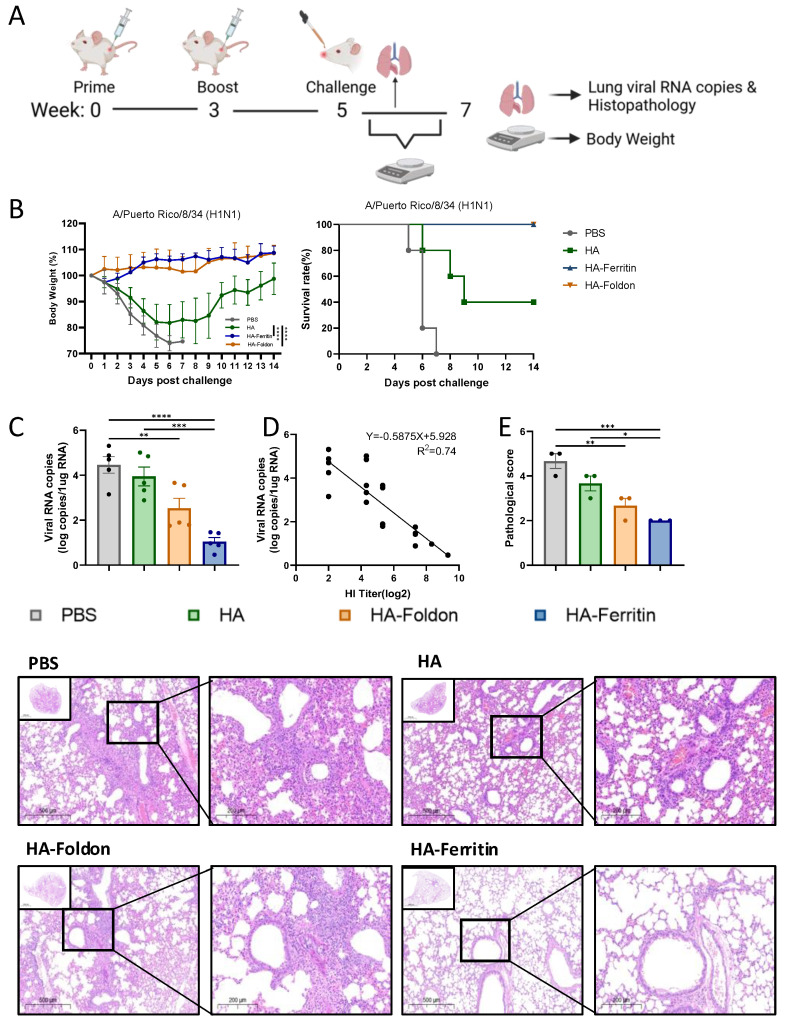
**Protective efficacy of vaccination against homologous viral challenge.** (**A**) Experimental timeline of immunization and viral challenge. BALB/c mice (*n* = 10 per group) were intramuscularly immunized with 25 μg of monovalent DNA vaccine (based on the H1N1 PR8 strain) delivered via electroporation. Two weeks post boost, mice were intranasally challenged with 20 μL of 10 LD_50_ homologous virus. Body weight and survival were monitored daily for 14 days post challenge (*n* = 5 for survival analysis). (**B**) Body weight changes (percentage of initial weight) and survival rates during the monitoring period. (**C**) Lung viral copy numbers quantified by absolute qPCR in lung tissues collected 5 days post challenge (*n* = 5). (**D**) Correlation of HI antibody titers with pulmonary viral loads was assessed through logarithmic transformation and linear regression modeling, with 95% confidence intervals for predicted values. (**E**) Representative H&E-stained lung sections from paraffin-embedded tissues (*n* = 3) and corresponding pathological scores. Scoring criteria: Grade 1, no lesions; Grade 2, <25% lobar involvement; Grade 3, 25–50%; Grade 4, 50–75%; and Grade 5, >75% involvement (perivascular/parenchymal infiltration). Higher scores indicate more severe pathology. All data points represent individual animals (mean ± SEM). Data are presented as mean ± SEM. Statistical significance was determined by one-way ANOVA: ns, not significant; * *p* < 0.05; ** *p* < 0.01; *** *p* < 0.001; and **** *p* < 0.0001.

To evaluate vaccine protective potential, we used the adaptive and lethal PR8 virus strain as the testing model to demonstrate the efficacy of each structural design. Mice with the PR8 vaccinated groups were intranasally challenged with 20 μL of 10 LD_50_ homologous PR8 virus at day 35 post booster immunization (Figure 4A). Fourteen-day monitoring revealed that the PBS control and HA-immunized groups exhibited significant weight loss accompanied by mortality within 5 days post challenge (DPI), with final survival rates of 0% and 40%, respectively, whereas HA-Foldon and HA-Ferritin vaccinated groups maintained stable body weights (Figure 4B). Quantitative RT-PCR analysis of lung tissues at 5DPI demonstrated that viral loads were reduced by one and two orders of magnitude in HA-Foldon and HA-Ferritin groups, respectively, while no significant difference was observed in the HA group compared to PBS controls (Figure 4C). Correlation analysis confirmed a significant negative relationship between HI antibody titers and pulmonary viral loads (r^2^ = 0.74) (Figure 4D). Histopathological analysis showed extensive inflammatory exudation with alveolar architecture destruction in the PBS control, focal inflammatory infiltration and vasculitis in the HA and HA-Foldon groups, and intact alveolar structure with minimal inflammation in the HA-Ferritin group (Figure 4E), which is consistent with viral load data and shows that while the HA-Ferritin vaccine provided near-complete protection, HA-Foldon conferred partial protective effects by mitigating but not completely preventing pathological progression.

### 3.5. Immunogenicity of Trivalent Seasonal Influenza Vaccines

**Figure 5:**
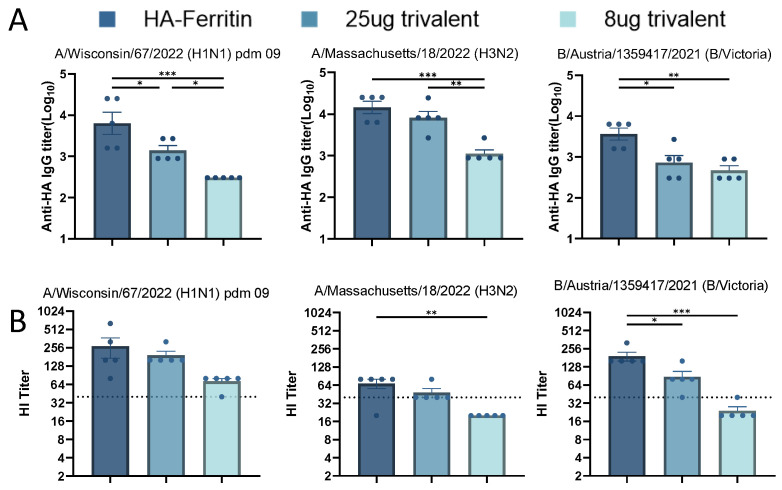
**Immunogenicity of trivalent seasonal influenza vaccines.** Based on monovalent HA-Ferritin vaccine groups (25 μg per HA subtype), two trivalent formulations were evaluated: (1) equal-dose trivalent (25 μg per HA subtype) and (2) low-dose trivalent (8 μg per HA subtype). Following the immunization schedule in Figure 2, serum samples collected 2 weeks post boost were analyzed for homologous strain-specific IgG antibodies by an ELISA (**A**) and hemagglutination inhibition (HI) titers (**B**). Data points represent individual animal measurements (mean ± SEM). Statistical comparisons were performed using one-way ANOVA: ns, not significant; * *p* < 0.05; ** *p* < 0.01; *** *p* < 0.001.

Initial studies demonstrated the promising immunogenicity of nanoparticle-designed HA-Ferritin vaccines. To systematically evaluate their potential as trivalent seasonal influenza vaccines, we formulated three vaccines by mixing plasmids encoding HA-Ferritin fusion proteins from H1N1, H3N2, and B influenza strains either with an equal-dose for the trivalent group (25 μg per HA subtype, total 75 μg) or a low-dose of the trivalent group (8 μg per HA subtype, total 24 μg). Serological analysis at day 35 post booster immunization revealed that the trivalent formulations elicited significantly lower binding antibody titers against the H1N1 and B subtypes compared to their monovalent counterparts, suggesting potential antigen competition or immunological interference (Figure 5A). The antibody response exhibited clear dose dependency, with further reduction observed in the low-dose group. We also assessed hemagglutination inhibition (HI) titers against the three seasonal influenza strains (Figure 5B). While the trivalent vaccine elicited comparable HI responses to H1N1 and H3N2, it induced significantly lower titers against the B strain. The low-dose trivalent formulation exhibited suboptimal immunogenicity across all three strains. These findings underscore critical considerations in multivalent vaccine design, particularly the dose-dependent efficacy and subtype-specific immune interference, which most notably compromised the response to influenza B.

## 4. Discussion

In this study, we developed three DNA vaccine constructs expressing seasonal influenza HA antigens: monomeric HA, trimeric HA incorporating a trimerization motif (HA-Foldon), and ferritin nanoparticle-displayed HA (HA-Ferritin). Stable trimeric structures were confirmed to be formed by both HA-Foldon and HA-Ferritin based on structural predictions and in vitro transfection experiments, while self-assembly into uniform nanoparticles of approximately 25 nm in diameter was observed for HA-Ferritin. Purified HA-Ferritin nanoparticles exhibited morphology consistent with previous reports [25]. Immunogenicity evaluation in murine models revealed no statistically significant differences in HA-specific binding antibody levels among the three constructs. While comparable in binding antibody induction, the constructs differed significantly in HI antibody responses, as evidenced by HA-Ferritin’s superior neutralizing antibody production against homologous seasonal influenza strains versus HA-Foldon and the poor performance of monomeric HA. Protective efficacy was further assessed through homologous challenge with PR8 virus in immunized mice. The HA-Ferritin and HA-Foldon vaccines provided near-complete protection. In contrast, monomeric HA showed markedly inferior protective efficacy (40% survival) compared to the trimeric formulations. The superior immunogenicity of HA-Ferritin aligns with recent advances in nanoparticle vaccine design. As shown in ferret studies [26], multivalent antigen presentation on ferritin nanoparticles enhances B-cell receptor crosslinking and germinal center responses, consistent with our observation of 3.27-fold lower viral loads compared to HA-Foldon. This mechanism is further supported by cryo-EM studies [27] of ferritin–HA complexes where ordered trimeric HA spikes mimic natural virion architecture, promoting conformational epitope exposure. However, the limited improvement in HI titers for H3N2 (Figure 2C) raises questions about subtype-specific structural constraints.

In recent years, the rapid advancement of vaccine technology has led to the development of multiple innovative platforms for seasonal influenza vaccines that overcome the technical limitations of conventional approaches, including mammalian cell-expressed recombinant subunit vaccines and mRNA vaccine platforms [28]. DNA vaccine platforms demonstrate distinctive advantages for influenza prevention and control: firstly, synthetic genetic engineering technologies enable significant reduction in vaccine development and production timelines, which is critical for meeting the urgent demands of pandemic response; and secondly, the absence of viral nucleic acids in DNA vaccines eliminates risks associated with live virus dissemination while substantially minimizing potential genomic integration, offering notable safety advantages. Our findings reinforce the unique value of DNA vaccines in influenza prevention. Compared to mRNA-LNP platforms requiring ultra-cold storage [29], DNA vaccines’ thermostability aligns with global distribution needs. Notably, the robust CD8+ T cell responses induced by HA-Ferritin (Figure 3) mirror results from H7N9 HA-DNA trials, where epitope-specific CTLs correlated with cross-protection [30]. This contrasts with protein subunit vaccines that predominantly elicit Th2-biased responses [31]. However, the dose-dependent efficacy decline in trivalent formulations (Figure 5) echoes challenges observed in multivalent mRNA vaccines [32], suggesting universal limitations in antigen competition that could be addressed through staggered immunization schedules or epitope focusing [33].

While HA-targeting neutralizing antibodies have been well-established as correlates of protection, T cell-mediated immune responses also play a pivotal role in viral control and clearance [34,35]. Previous studies have demonstrated that T cell responses alone can confer protection against influenza virus in mice even in the absence of neutralizing antibodies [36]. Our study reveals that DNA vaccines not only elicit humoral immunity but also induce robust expansion of high-quality IFN-γ-secreting T cells. In vitro peptide stimulation assays showed that vaccination against the H3N2 strain induced significantly higher frequencies of IFN-γ-producing cells compared to H1N1-targeted immunization. Notably, although the H3N2 vaccine elicited relatively lower HI antibody titers, it still provided partial protection through the activation of cellular immune responses [37], suggesting that T cell immunity serves as an important complementary mechanism in influenza protection.

Substantial differences exist among influenza virus subtypes in their capacity to induce functional antibody responses. Our study demonstrates that while vaccines targeting seasonal H3N2 strains elicit HA-specific IgG antibody levels comparable to those induced by H1N1 vaccines, they generate significantly lower hemagglutination inhibition (HI) antibody titers. This phenomenon shows remarkable consistency with the H3N2-specific immune profiles observed in trivalent influenza vaccines developed using either recombinant protein technology [38] or mRNA platforms [32]. Notably, similar suboptimal HI antibody responses have been documented for H7 subtype influenza, where both natural infection [33] and inactivated vaccination [39,40] consistently induce lower serum antibody titers. Current evidence suggests these subtype-specific disparities may stem from intrinsic structural characteristics of viral HA proteins, particularly the distinctive conformational features of receptor-binding sites (RBSs) that could create steric hindrance for antibody binding, thereby compromising neutralizing potency [41]. Recent work on H3 HA glycosylation patterns suggests that steric hindrance from conserved glycan shields may partially explain this disparity, warranting glycoengineering strategies in future designs [42].

While the vaccine strategy proposed in this study shows promising potential, several limitations should be acknowledged. Although we observed high levels of hemagglutination inhibition activity against seasonal influenza strains in murine models, protective efficacy challenge experiments in ferret models have not yet been conducted. Therefore, the immune protective capacity of this vaccine in other animal models and ultimately in human populations requires further evaluation.

It is important to consider the potential for hepatotoxicity when evaluating the safety profile of any vaccine, particularly those that may induce systemic inflammatory responses. However, unlike mRNA vaccines formulated with lipid nanoparticles (LNPs), which are known to accumulate predominantly in the liver following systemic administration [43], DNA vaccines are typically delivered as naked plasmids and remain largely localized at the site of injection—most commonly the skeletal muscle if intramuscular injection is employed [44]. This distinct biodistribution pattern significantly limits direct exposure of the liver to the vaccine construct, thereby reducing the likelihood of hepatic toxicity. Moreover, numerous preclinical [45] and clinical studies [46] on DNA vaccines have consistently demonstrated a favorable safety profile with no significant elevation in liver enzymes such as ALT, AST, ALP, or bilirubin, further supporting the low risk of hepatotoxicity associated with this platform. These findings align with the established behavior of plasmid DNA-based immunization strategies and provide additional confidence in the safety of our ferritin-based HA DNA vaccine candidate.

## 5. Conclusions

In summary, DNA vaccines represent a promising platform to overcome the limitations of current influenza vaccine technologies. Our findings demonstrate that nanoparticle-displayed antigen vaccines can induce potent strain-matched hemagglutination inhibition (HI) activity and enhance subtype-specific HI efficacy in murine models. Furthermore, DNA vaccines elicit high-quality T cell immune responses. The combined strategies of trimerization motif stabilization and ferritin-based nanoparticle display not only advance influenza vaccine development but also hold potential for application against other highly mutable pathogens.

## 6. Patents

Some of the results from the work reported in this manuscript has been used for a patent application.

## Data Availability

Data are contained within the article.

## Supplementary Materials

The following supporting information can be downloaded at: https://www.mdpi.com/article/10.3390/vaccines13070745/s1, Figure S1: In vitro characterization of influenza HA antigens; Figure S2: HA-specific IgG1 and IgG2a immune responses.
