# Supplementary material for: Ferritin-Based HA DNA Vaccine Outperforms Conventional Designs in Inducing Protective Immunity Against Seasonal Influenza
Source: Vaccines (Basel). 2025 Jul 10;13(7):745. doi: 10.3390/vaccines13070745 (PMC12299999; doi:10.3390/vaccines13070745)
Supplement: Supplementary file 1 [file vaccines-13-00745-s001.zip › vaccines-3715154-supplementary.pdf]

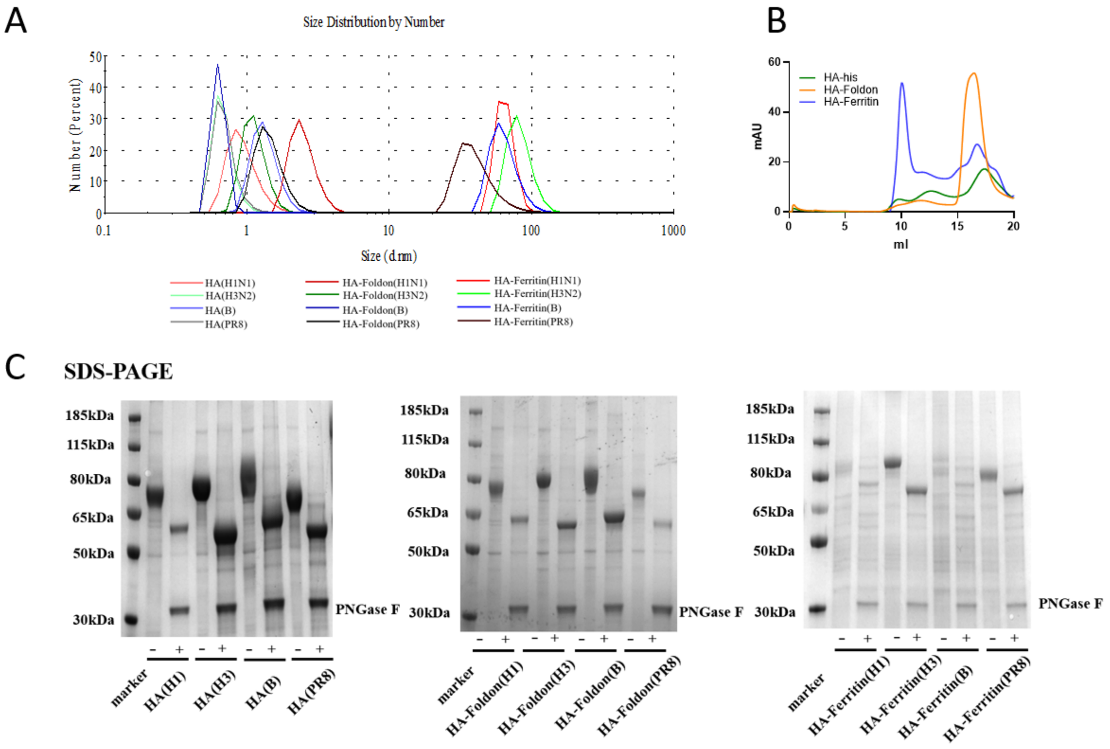

Figure S1. In vitro characterization of influenza HA antigens.

(A) Particle size distribution analysis by DLS. Purified HA, HA-Foldon, and HA-Ferritin proteins were subjected to DLS to evaluate particle homogeneity and size characteristics. Each sample was independently measured in triplicate.

(B) SEC profiles of proteins. The elution profiles of HA-His, HA-Foldon, and HA-Ferritin were monitored by UV absorbance at 280 nm. The peak positions and shapes reflect the molecular weights and oligomeric states of the proteins.

(C) Glycosylation analysis by SDS-PAGE. Purified HA, HA-Foldon, and HA-Ferritin proteins were treated with PNGase F to remove N-linked glycans, followed by SDS-PAGE analysis. The migration shifts before and after deglycosylation demonstrate the glycosylation status and its impact on protein electrophoretic mobility.

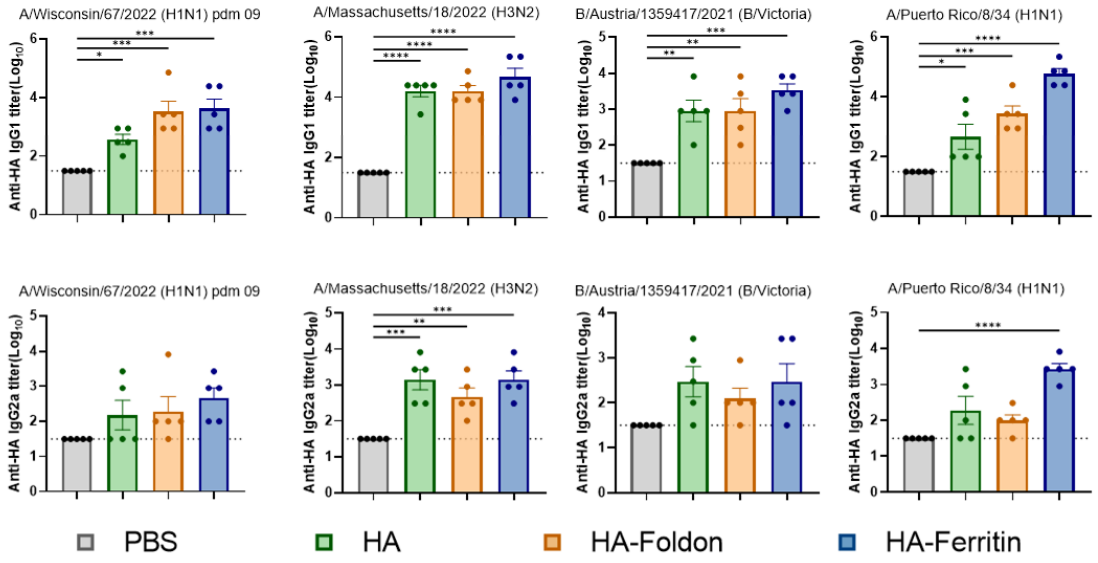

Figure S2. HA-specific IgG1 and IgG2a immune responses.

Serum levels of HA-specific IgG1 and IgG2a antibodies against the homologous viral strain were measured by ELISA two weeks after the booster immunization, characterizing the isotype profile of vaccine-induced humoral immune responses. Individual data points represent measurements from separate animals, expressed as mean ± SEM. Statistical significance between groups was determined by one-way ANOVA with the following thresholds: ns, not significant; *p<0.05; **p<0.01; ***p<0.001; ****p<0.0001.

Supplementary Materials

Dynamic Light Scattering (DLS)

Protein samples were centrifuged at 10,000 × g for 10 min at 4°C to remove aggregates, and the supernatants were filtered through 0.22-μm membranes. For analysis, 40 μL of each sample was loaded into a microcuvette, and particle size distribution was measured at 25°C using a Zetasizer Nano Series instrument (Malvern Panalytical, Malvern, UK, ZS90) with three sequential scans per sample.

Size-Exclusion Chromatography Purification of Protein Using Superose 6 Increase Column

The protein sample was purified by size-exclusion chromatography on a Superose 6 Increase 10/300 column (Cytiva, Marlborough, MA, USA) using an ÄKTA pure system (Cytiva, Marlborough, MA, USA). Prior to loading, the column was equilibrated with at least 2 column volumes (CV) of PBS running buffer. The clarified protein sample was loaded at a flow rate of 0.5 mL/min. The elution profile was monitored at 280 nm, and peak fractions corresponding to the target protein were pooled based on their elution volume. The pooled fractions were concentrated using an appropriate molecular weight cut-off centrifugal filter and analyzed by SDS-PAGE for purity assessment. The

column was regenerated with 1 CV of running buffer and stored in 20% ethanol at 4°C for long-term preservation.

PNGase F-Mediated Deglycosylation and SDS-PAGE Analysis of Glycoproteins

Protein samples were denatured in Denaturing Buffer at 100°C for 10 min, followed by incubation with Glyco Buffer containing 10% NP-40 and PNGase F (New England Biolabs, Ipswich, MA, USA; Cat. #P0704) at 37°C for 60 min to complete deglycosylation.
After enzymatic treatment, samples were mixed with SDS-PAGE loading buffer and heated at 100°C for 10 min. The samples were then separated on 4–12% Bis-Tris gradient gels using MOPS running buffer at 160 V for 60–70 min until the dye front reached the bottom of the gel. Proteins were visualized by staining with InstantBlue Coomassie.

Serum IgG1 and IgG2a ELISA Analysis Post-Immunization

Serum samples were collected from mice two weeks after secondary immunization, centrifuged at 3000×g for 10 min to remove debris. Antigen immobilization was performed by coating high-binding 96-well plates (Corning, New York, NY, USA) with target proteins at 2 μg/mL in carbonate buffer (pH 9.6) for 16 h at 4°C. After three washes with PBST, plates were blocked with 5% skim milk at 37°C for 1 h. Serum samples were then subjected to two-fold serial dilutions (starting from 1:100) in 2% skim milk and incubated with immobilized antigens for 90 min at 37°C. Following three washes with PBST, plates were incubated with rat anti-mouse IgG2a (Southern Biotech, Birmingham, AL, USA, Cat. #1155-05) or rat anti-mouse IgG1 (Southern Biotech, Birmingham, AL, USA, Cat. #1144-05) antibodies at 37°C for 1 h. Immunoreactivity was visualized using 3,3',5,5'-tetramethylbenzidine (TMB) substrate (TianGen, Beijing, China) for precisely 15 min at room temperature. The enzymatic reaction was terminated by acidification with 1 M sulfuric acid and absorbance was measured at 450/620 nm using a microplate reader (Bio-Rad, Hercules, CA, USA).
